# Immune Dysfunctions and Immune-Based Therapeutic Interventions in Chronic Lymphocytic Leukemia

**DOI:** 10.3389/fimmu.2020.594556

**Published:** 2020-11-18

**Authors:** Valentina Griggio, Francesca Perutelli, Chiara Salvetti, Elia Boccellato, Mario Boccadoro, Candida Vitale, Marta Coscia

**Affiliations:** ^1^ University Division of Hematology, A.O.U. Città della Salute e della Scienza di Torino, Torino, Italy; ^2^ Department of Molecular Biotechnology and Health Sciences, University of Torino, Torino, Italy

**Keywords:** chronic lymphocytic leukemia, immune dysfunction, immunotherapy, immunomodulation, targeted therapy, cellular therapy, chimeric antigen receptor T cells

## Abstract

Chronic lymphocytic leukemia (CLL) is a B-cell malignancy characterized by a wide range of tumor-induced alterations, which affect both the innate and adaptive arms of the immune response, and accumulate during disease progression. In recent years, the development of targeted therapies, such as the B-cell receptor signaling inhibitors and the Bcl-2 protein inhibitor venetoclax, has dramatically changed the treatment landscape of CLL. Despite their remarkable anti-tumor activity, targeted agents have some limitations, which include the development of drug resistance mechanisms and the inferior efficacy observed in high-risk patients. Therefore, additional treatments are necessary to obtain deeper responses and overcome drug resistance. Allogeneic hematopoietic stem cell transplantation (HSCT), which exploits immune-mediated graft-*versus*-leukemia effect to eradicate tumor cells, currently represents the only potentially curative therapeutic option for CLL patients. However, due to its potential toxicities, HSCT can be offered only to a restricted number of younger and fit patients. The growing understanding of the complex interplay between tumor cells and the immune system, which is responsible for immune escape mechanisms and tumor progression, has paved the way for the development of novel immune-based strategies. Despite promising preclinical observations, results from pilot clinical studies exploring the safety and efficacy of novel immune-based therapies have been sometimes suboptimal in terms of long-term tumor control. Therefore, further advances to improve their efficacy are needed. In this context, possible approaches include an earlier timing of immunotherapy within the treatment sequencing, as well as the possibility to improve the efficacy of immunotherapeutic agents by administering them in combination with other anti-tumor drugs. In this review, we will provide a comprehensive overview of main immune defects affecting patients with CLL, also describing the complex networks leading to immune evasion and tumor progression. From the therapeutic standpoint, we will go through the evolution of immune-based therapeutic approaches over time, including i) agents with broad immunomodulatory effects, such as immunomodulatory drugs, ii) currently approved and next-generation monoclonal antibodies, and iii) immunotherapeutic strategies aiming at activating or administering immune effector cells specifically targeting leukemic cells (e.g. bi-or tri-specific antibodies, tumor vaccines, chimeric antigen receptor T cells, and checkpoint inhibitors).

## Introduction

Chronic lymphocytic leukemia (CLL) is a lymphoproliferative disease characterized by the clonal accumulation of mature B lymphocytes in the peripheral blood, bone marrow and secondary lymphoid organs (1). A hallmark of CLL is the variable clinical course, which reflects the biological heterogeneity of tumor cells. The lack of somatic mutations on immunoglobulin heavy chain variable (IGHV) genes, and/or the presence of chromosomal aberrations and genetic lesions identify patients with more aggressive forms of the disease [as reviewed in (1, 2)]. Besides intrinsic features of the malignant clone, profound defects of the immune system and the ability of leukemic cells to circumvent immune recognition and elimination are leading causes of tumor progression. In CLL, tumor cells and cellular components of the microenvironment are reciprocally interconnected and co-evolve, shaping each other during the course of the disease [as reviewed in (3–5)].

Some immunological alterations [e.g. T-cell and natutal killer (NK)-cell expansion, and reduction of circulating normal B cells] are also associated with monoclonal B-cell lymphocytosis (MBL), a premalignant condition that precedes CLL (6, 7). However, most of the immune surveillance dysfunctions accumulate during disease evolution, most likely constributing to the transition from MBL to CLL [as reviewed in (8, 9)]. From the clinical standpoint, these immunologic dysregulations are responsible for the increased susceptibility to infections and secondary malignancies, the occurrence of autoimmune phenomena and the failure to control disease progression (10, 11) [and as reviewed in (5, 12–17)].

In patients with CLL carrying favorable prognostic factors (i.e. mutated IGHV genes), the chemoimmunotherapy combination regimen consisting of fludarabine, cyclophosphamide, and rituximab (FCR) allows the achievement of undetectable minimal residual disease and long-term remissions (18). More recently, combinations including multiple targeted drugs with a different mechanism of action, such as BTK and Bcl-2 inhibitors with or without anti-CD20 monoclonal antibodies (mAb), have shown very promising results in terms of depth and durability of response, although data are not yet mature (19–21).

Nevertheless, to date, the only therapeutic approach with a consolidated, long-term potentially curative effect is the allogeneic hematopoietic stem cell transplantation (HSCT). HSCT, which is still considered a valuable treatment option for younger and fit patients with high-risk CLL (i.e. relapsed/refractory patients with poor prognostic features), exploits a T-cell mediated graft-*versus*-leukemia reaction, thus supporting the evidence that a competent immune system can be effective in controlling and eradicating the tumor. Due to the advanced median age and the high frequency of comorbidities, HSCT can be reserved only to a restricted number of patients with CLL [as reviewed in (22)]. However, other approaches exploiting immunological mechanisms, such as adoptive chimeric antigen receptor (CAR) T-cell therapy, possibly in combination with drugs showing immunomodulatory properties (e.g. lenalidomide and targeted drugs), have shown promising preclinical and/or clinical results.

In this review, we will comprehensively describe immune alterations occurring in CLL, and we will go through the evolution of immune-based therapeutic approaches over time, also addressing most recent advances in the field of immunotherapy.

## Immune Escape in CLL

**Figure 1:**
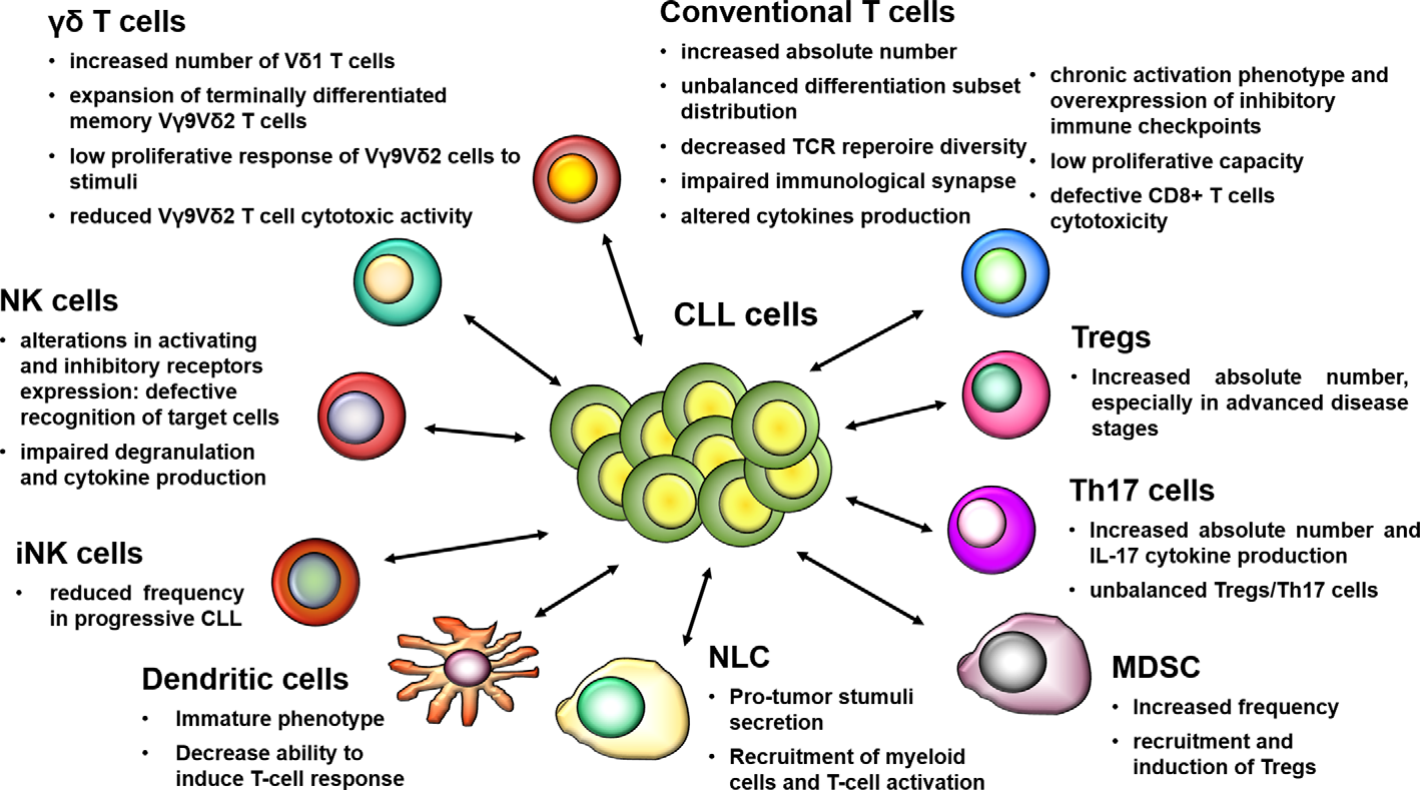
Schematic overview of main defects affecting immune cell populations in CLL. The dysregulation of the immune response in CLL includes phenotypic alterations and functional impairments, which are present since the early stages and exacerbate during the course of the disease, thus promoting immune tolerance and tumor progression. The mutual interactions between leukemic cells and cellular elements of the immune system contribute to the establishment of a permissive or even supportive microenvironment that favors tumor progression, thus playing a key role in immune escape mechanisms.

During the clinical course, CLL cells induce a progressive impairment of the immune system leading to a state of clinically manifest immune suppression, which is in part responsible for the lack of disease control [as reviewed in (3, 4)]. The association between immune deficiency and tumor progression has been widely explored in CLL. Studies report that immune defects characterizing patients since diagnosis frequently exacerbate in advanced CLL stages, and that a wide range of quantitative and qualitative alterations affect both the innate and the adaptive arms of the immune response, have been reported (as summarized in **Figure 1**) [as reviewed in (5, 23, 24)]. Defects in main players of innate immunity, which include cell populations of lymphoid (i.e. NK cells, NKT cells, and γδ T cells) and myeloid [i.e. dendritic cells (DC) and macrophages] lineage, contribute to the ineffective triggering and maintenance of T-cell responses, as well as to their suboptimal cytotoxic activity. In the context of the adaptive immune response, several aberrations of the T-cell compartment, ranging from phenotypical changes to functional impairment, have been described [as reviewed in (24–26)]. Besides cellular components, significant alterations of the humoral response also contribute to the tumor immune escape in CLL (5). Elucidating the immune cell dysfunctions and identifying the mechanisms underlying immune suppression are crucial steps to attempt an immune system reactivation, and develop effective and novel immune-based treatment strategies.

### Phenotypic and Functional Alterations of the NK- and NKT-Cell Compartments

Most studies report that NK-cell number is increased in the peripheral blood of CLL patients and associates with better prognosis (27–34). NK cells represent an appealing lymphocyte subset to be exploited in the context of immunotherapy, especially for their non-major histocompatibility complex (MHC)-restricted cytotoxicity (35). However, it has been demonstrated that autologous NK cells are not able to effectively eliminate CLL cells, because of both NK-cell intrinsic defects and immune escape mechanisms employed by tumor cells (36–41). The effector function of NK cells rely on combined signaling *via* a variety of activating and inhibitory receptors whose ligands are either expressed on the surface of target cells or secreted in soluble forms. Several studies focused their attention on the balance between inhibitory and activating receptors signals in CLL, and their role in regulating the final NK-cell-mediated anti-tumor response. The inhibitory receptors NKG2A and the killer-cell immunoglobulin-like receptors (KIRs), through the binding with their respective ligands HLA-E and HLA-A on tumor cells, suppress cytokine secretion and hamper direct cytotoxicity of NK cells against target cells (31, 42). The expression of NKG2A is similar on NK cells from CLL patients and healthy donors, whereas its ligand HLA-E is overexpressed on the surface of leukemic cells (41–44). It has been reported that plasma levels of soluble HLA-E (sHLA-E) are higher in advanced-stage CLL patients and associate to shorter treatment free survival. In addition, sHLA-E secreted by tumor cells *in vitro* inhibits cell degranulation and IFN-γ production by NK cells, thus determining their functional impairment (44). Similarly, plasma samples from CLL patients were reported to contain increased levels of soluble HLA-G, the ligand of the inhibitory receptor (KIR)2DL4, and to be capable of dampening both the viability and cytotoxic function of NK cells from healthy donors *in vitro* (45). HLA-G is also bound with high affinity by the Ig-like transcript 2 (ILT2) inhibitory receptor, which is overexpressed on NK cells from CLL patients (43). As an additional inhibitory mechanism, in line with data on conventional T cells, the immune checkpoint Tim-3 was found to be aberrantly expressed on the NK-cell compartment (28). Concerning activating receptors, the reduced expression of NKG2D, DNAM-1 and natural cytotoxicity receptors (NCRs) reported on NK cells of CLL patients compared to healthy individuals, is paralleled by a defective cytotoxic activity, degranulation and direct killing of target cells (28, 31, 32, 41, 46, 47). Of note, CLL cells have decreased surface level of NKG2D and NCRs ligands, which are also shed as soluble molecules (i.e. sMIC-A, sMIC-B, and sULBP2), thus contributing to a hindered recognition of tumor cells by NK cells (48–50). Notably, NK-cell dysfunctions are not permanent and can be reversed by proper stimulation with cytokines (i.e. IL-2, IL-15, IL-27) (41, 51, 52).

Despite the abnormalities reported so far, NK cells retain their ability to efficiently induce antibody-dependent cellular cytotoxicity (ADCC), through the binding of CD16 (FcγRIIIA) to the Fc-regions of antibody-antigen complexes located on the surface of tumor cells (31, 34, 41, 46, 53). In CLL, ADCC has a pivotal therapeutic role because several treatment strategies include anti-CD20 mAb, whose activity rely on this process. Due to their preserved ADCC function and the reversibility of other CLL-related dysfunctions, NK cells are therefore an attractive source for cellular immunotherapy in this disease.

Within innate immunity, another cell player with a potential anti-tumor role are type I NKT cells, also called invariant NKT (iNKT) cells. iNKT cells have the ability to activate and expand in response to antigens presented by CD1d (54–57). In CLL, little information regarding NKT cells and, specifically, iNKT cells is currently available, and mainly supports their contribution to CLL immune surveillance (58, 59). Interestingly, iNKT-cell frequency is significantly lower in patients with progressive disease than in patients with stable disease, and has shown to be an independent predictor of disease progression (60). Concerning the leukemic counterpart, a reduced expression of CD1d has been described on CLL cells compared to normal B cells from healthy donors (58, 59, 61, 62). From the functional standpoint, CLL cells have a limited ability to present glycolipid antigens to iNKT cells and to induce their expansion and functional activation (58, 63, 64). Of note, this reduced capacity of leukemic cells to stimulate iNKT cells can be effectively reversed by retinoic acid, which upregulates the expression of CD1d on CLL cells and enhances iNKT-mediated cytotoxicity against tumor targets loaded with α-galactosylceramide (59).

### γδ T-Cell Alterations

Among various lymphocyte subsets being considered for cellular immunotherapy of cancer are γδ T cells (both Vδ1- and Vδ2-expressing T cells), which have the ability to mediate responses through the activation of cytotoxic mechanisms against tumor cells in a MHC-unrestricted manner (35). Patients with CLL have increased numbers of circulating Vδ1 T cells which are able to produce TNF-α and INF-γ. Moreover, Vδ1 T cells are able to kill leukemic cells expressing the MIC-A and ULBP3 surface molecules, which are involved in the activation of γδ T-cell effector functions through the engagement of the NKG2D receptor (65). Interestingly, preclinical studies reported that Vδ1 T cells can be properly stimulated to express NCRs that act as costimulatory molecules and improve their cytotoxic activity against CLL cells both *in vitro* and in a xenograft mouse model (66, 67). In healthy subjects, the main subset of circulating γδ T cells is represented by Vγ9Vδ2 T cells, which consist of cytotoxic T lymphocytes with a putative potent anti-tumor activity, triggered by the MHC-independent recognition of non-peptidic phosphoantigens, such as intermediate metabolites of the mevalonate metabolic pathway and aminobisphosphonates [as reviewed in (68)]. We have previously demonstrated that in CLL the Vγ9Vδ2 T cell-compartment is characterized by an unbalanced differentiation subset distribution, with a prominent expansion of effector memory and terminally differentiated effector memory cell subsets, determining a low *in vitro* proliferative response and predicting for a more aggressive clinical course (69). In addition, de Weerdt *et al*. reported that Vγ9Vδ2 T cells from CLL patients are less effective in inducing tumor cell death, due to a dysfunction in effector cytokine production and degranulation. Interestingly, the observations that an altered phenotype is also inducible in healthy Vγ9Vδ2 T cells co-cultured with CLL cells, and that in CLL patients the functional impairment of Vγ9Vδ2 T cells is associated with higher leukocytes counts, indicate a leukemia-induced mechanism of immune suppression (70).

### DC Defects

DC are specialized antigen presenting cells (APC) with a crucial role in the initiation and regulation of innate and adaptive immune responses, and whose functional modulation is under investigation with the aim of improving cancer immunotherapy. In CLL, DC show an immature phenotype (lack of CD80 and CD83 expression), and have an altered capacity to stimulate T-cell proliferation, to drive T-cell differentiation toward T helper (Th)1 response and to release IL-12 (71, 72). In addition, Orsini *et al.* reported that CLL cells are able to modify the maturation and function of healthy donor-derived DC through the secretion of IL-6 (71). Recent data demonstrated that the molecular mechanism underlying DC abnormalities in CLL is a disruption of the IL-4R/STAT6 pathway due to enhanced levels of the suppressor of cytokine signaling 5 (SOCS5), a negative regulator that inhibits STAT6 activation and leads to a defective DC differentiation (72). Notably, despite these observed alterations, different studies and clinical trials showed that DC from CLL patients can be properly manipulated and effectively exploited in the context of vaccination approaches (73–77).

### Phenotypic and Functional T-Cell Alterations

T lymphocytes have a fundamental role in tumor immune-surveillance. In the context of adaptive immune response, CD4+ Th cells are the main actors in antigen recognition, activation of humoral response, cytokine production, and coordination of CD8+ cytotoxic T lymphocyte response [as reviewed in (25, 26)]. Overall, circulating CD4+ and CD8+ T lymphocytes are increased in patients with CLL (78–80). The expansion of CD8+ T cells is prominent and results in a drop of the CD4:CD8 ratio, which characterizes CLL patients since the early phases of the disease (79, 81). A number of studies attributed a prognostic value to the CD4:CD8 ratio, whose inversion has been associated with advanced disease, and has shown to predict a shorter time to first treatment and overall survival (82–84). Concerning Th subset distribution, most reports agree on the accumulation of Th1 T cells in the peripheral blood of CLL patients compared to healthy controls, whereas data on Th2 T cells are still controversial (85–88). From the functional standpoint, a recent article by Roessner *et al*. has investigated the pro‐ or anti-tumoral effect of Th1 T cells on CLL development, showing that the accumulation of Th1 T cells observed in human CLL and in a mice with CLL‐like disease has no impact on disease progression (87). In terms of T-cell differentiation subsets distribution, a reduction in naïve T cells and an accumulation of effector T cells and highly differentiated memory T cells were observed in CLL patients (82, 85, 89, 90). Several evidences suggest that T lymphocytes in CLL patients are subjected to chronic antigenic stimulation, which shapes their phenotype and functional activity. Indeed, T cells show an increased surface expression of CD57, CD69, and HLA-DR, which are typical markers of activated cells (91, 92). Phenotypic and functional properties of T cells from patients with CLL resemble those of exhausted T cells, which are typically observed during chronic infections (93). Although cytomegalovirus (CMV) infection has shown to induce T-cell expansion and modulate the distribution of differentiation subsets, the exhaustion observed in T cells from CLL patients resulted to be independent from CMV serostatus (82, 93–95). The progressive skew of the T-cell receptor (TCR) repertoire occurring during disease progression, may suggest a tumor-related antigen-mediated selection (96, 97). In line with this observation, an oligoclonal CD8+ effector T-cell population, that expands along with CLL progression and controls disease development, was observed in both CLL patients and mice bearing a CLL-like disease (90). Consistently, through a cell-to-cell-mediated mechanism, leukemic cells induce in CD4+ and CD8+ T cells purified from CLL patients several changes in the expression of genes involved in CD4+ T-cell differentiation, cytoskeleton formation, and vesicle trafficking, and in CD8+ T-cell cytotoxicity (98). These evidences confirm the contribution of the leukemic counterpart in shaping a pro-tumor microenvironment. The aberrant gene expression profile has an impact on the T-cell functions, mainly in terms of immunological synapse formation with APC, proliferation, migration, and cytotoxic activity (99–102). In particular, a key regulatory mechanism of immune evasion in CLL is the impaired killing of target cells by cytotoxic T lymphocytes, which is associated with the formation of dysfunctional non-lytic immune synapses and to a non-polarized release of lytic granules (101). Lastly, also metabolic features, such as mitochondrial respiration, membrane potential and levels of reactive oxygen species, have an impact on T-cell fitness, and demonstrate to be particularly relevant for CAR T-cell expansion and persistence (103).

The aberrant expression of immune checkpoint molecules, which regulate T-cell activation and function, is a hallmark of an impairment in immune surveillance. The engagement of checkpoint receptors by their ligands leads to the inhibition of T-cell proliferation and cytokine production, thus suppressing immune responses. In CLL, both CD4+ and CD8+ T cells show an increased expression of several inhibitory checkpoints, such as CTLA-4, PD-1, LAG3, Tim-3, TIGIT, CD160, and CD244 (89, 93, 104–110). Of note, this abnormal expression of immune checkpoint receptors on T lymphocytes is paralleled by an increased expression of their corresponding inhibitory ligands, such as PD-L1/PD-L2, CD200, galectin-9, and CD276, on leukemic cells (89, 99, 110, 111). CTLA-4 and PD-1 are the more extensively studied immune checkpoints in CLL. T cells from CLL patients have a higher expression of both the intracellular and surface forms of CTLA-4 compared to healthy controls (85, 112). In addition, the upregulation of PD-1 was observed in CD4+ and CD8+ T cells from patients with CLL and was reported to associate with adverse prognosis (82, 85, 89, 113). PD-1 expression is further increased in T cells from the lymphnode compared to the peripheral blood compartment (90, 114). Interestingly, the double positivity for PD-1 and Tim-3 identifies a T-cell subset with a particularly pronounced impairment in the effector functions (108). Despite their features of impairment, some functional aspects of T cells from the peripheral blood of CLL patients, such as cytokine production, were initially reported to be preserved or even enhanced in comparison to healthy individuals (93). By contrast, CD8+ T cells from secondary lymphoid organs, which are continuously exposed to leukemic cells, express higher levels of PD-1 and are functionally defective (90, 114).

A better understanding of the mechanisms leading to this tumor-induced dysfunction of CD8+ T cells will be important for the development of effective T-cell-based immunotherapeutic strategies for the treatment of CLL patients.

### Features of Immunosuppressive Cells and of the Tolerogenic Milieu in CLL

Multiple signals emanated by tumor cells shape the tumor supportive functions of different cellular elements of the tumor microenvironment, including stromal cells, T cells, and myeloid-derived cells. In addition, extrinsic features of the tumor niche—such as hypoxia—contribute to this tolerogenic milieu, leading to the engagement of cell subsets endowed with immune suppressive properties, such as T regulatory cells (Tregs) and myeloid-derived suppressor cells (MDSC) (115).

Several studies agree with the demonstration that circulating Tregs count is increased in CLL patients with respect to healthy controls, while data on Tregs frequency among total CD4+ T cells are not consistent (116–119). Higher Tregs number associates with increased tumor load, advanced stages of disease, disease progression, and poor prognosis (116, 117, 120, 121). Interestingly, Jak *et al*. reported that the expansion of Tregs in CLL can be mediated by CD27-CD70 interactions in the lymphnode and by an impaired sensitivity to apoptosis linked to a Bcl-2 overexpression, rather than being the consequence of chronic antigenic stimulation (122). In parallel to the higher number, an increased production of IL-10 and TGF-β1, and an overexpression of the immunosuppressive molecule CTLA-4 characterize Tregs from CLL patients compared to controls (104, 121, 123). From the functional standpoint, available information indicates that the suppressive activity of Tregs is preserved in patients with CLL (117). Indeed, Tregs i) proliferate in response to TCR stimulation, ii) display CD107a surface expression as a marker of cytotoxic activity, and iii) are able to inhibit T-cell proliferation (120, 124, 125). In addition, a recent study has demonstrated that in CLL patients Tregs switch toward an effector-like T-cell phenotype, which is associated with an altered cytokine profile and transcriptional program of immune genes (126).

Tregs are strictly interconnected with the Th17-cell population, a subset of pro-inflammatory cells involved in the development and evolution of tissue inflammation and autoimmune diseases, and with a dichotomous role in cancer [as reviewed in (127)]. Patients with CLL have an increased frequency and absolute number of Th17 cells, and higher IL-17A and IL-17F serum levels compared to healthy controls. Of note, the Th17-cell number is associated with the presence of favorable prognostic factors, an early stage of the disease and a longer overall survival (85, 88, 128–133). Although these evidences suggest that Th17 cells may have a protective function, their role within the CLL microenvironment is not yet fully understood. Indeed, a recent study by Zhu *at el*. reported that the stimulation of CLL cells and bone marrow mesenchymal stem cells with IL-17 induces the generation of IL-6, a pro-survival cytokine for tumor cells. Consistently, results obtained by *in vivo* experiments have demonstrated that IL-17 treatment favors CLL cells engraftment in a xenograft model through an IL-6-mediated mechanism (134). Lastly, a rise in the Th17-cell count leading to an unbalanced Tregs/Th17 ratio has been found in a subset of patients who undergo autoimmune complications, suggesting a possible contribution of this cell population to the pathogenesis of CLL-related autoimmune cytopenias [as reviewed in (128)].

Another player in the tolerogenic milieu of CLL is represented by MDSC, which accumulate in the peripheral blood of CLL patients. MDSC are defined as CD14+/HLA-DR^low^ cells and are endowed with potent immunosuppressive activity, limiting the T-cell-mediated anti-tumor responses and the effectiveness of immune therapeutic approaches [as reviewed in (135, 136)]. The frequency of MDSC associates with tumor progression and poor prognosis in CLL (137–140). Different studies provided the evidence of a complex network of interactions occurring between tumor cells, T-cell compartments, and MDSC. CLL cells enhance indoleamine 2,3-dioxygenase (IDO) gene expression in MDSC, which, in turn, suppresses *in vitro* T-cell activation and induces suppressive Tregs (138). On the other hand, MDSC depletion results in a control of CLL development and restores T-cell differentiation subsets skewing in the Eµ-TCL1 mouse model, thus confirming the role of MDSC in mediating CLL-related immune dysfunctions (141). An additional mechanism by which CLL cells maintain a supportive microenvironment is the secretion of exosomes inducing PD-L1 and inflammatory cytokine expression in monocytes, thus triggering their reprogramming toward MDSC (142, 143).

Among cells of myeloid origin, nurse like cells (NLC) are known to generate *in vitro* from monocytes and to contribute to CLL cells protection from spontaneous and drug-induced apoptosis. The *in vivo* counterpart of NLC is represented by tumor-associated M2-polarized macrophages, typically residing in the lymphnode and bone marrow of CLL patients. NLC actively shape the microenvironment through the secretion of several cytokines and chemokines: on one hand, NLC attract tumor cells and sustain their survival and, on the other, they promote the recruitment of accessory myeloid cells and stimulate T-cell activation and proliferation (136, 144).

### Humoral Immune Response

Up to date, there are only few data regarding the antibody-mediated responses and healthy B-cell compartment in CLL, and most of them focus on immunoglobulin (Ig) deficiencies. Hypogammaglobulinemia is a very common feature and is associated with an increased risk of infections, which largely contributes to morbidity and mortality of CLL patients. At diagnosis, up to 60% of patients has decreased levels of serum Ig, with IgG, IgA, and IgM as the most affected Ig classes (145). It is well established that hypogammaglobulinemia severity correlates with advanced disease stage and worsens during progression, also correlating with shorter time to first treatment (146–148). The mechanism causing hypogammaglobulinemia is not completely clear, but it could be related to a CLL-mediated inhibition of polyclonal antibodies production and to a reduction in the number of healthy B cells (149, 150).

Another consequence of the altered humoral immunity is the occurrence of autoimmune complications. Episodes of autoimmune cytopenia are frequently observed in CLL patients and are attributable to high affinity polyclonal IgG auto-antibodies which are produced by the non-leukemic B cells and target membrane antigens expressed on red blood cells, platelets, or granulocytes [as reviewed in (17, 151, 152)]. In addition, we previously reported that polyclonal antibodies directed to recurrent antigens expressed by tumor cells can be frequently found in the serum of CLL patients, especially those with progressive disease. However, these antibodies are inefficient in triggering ADCC and complement-derived cytotoxicity (CDC) (153). Alterations in the classical components of the complement cascade are reported in almost 40% of CLL patients, and contribute to the compromised CDC and to their increased susceptibility to infections (154, 155). Of note, complement defects may also impair the clinical efficiency of anti-CD20 mAb, which at least partially rely on CDC for their activity.

## Immunomodulation and Immunotherapy in CLL

**Figure 2:**
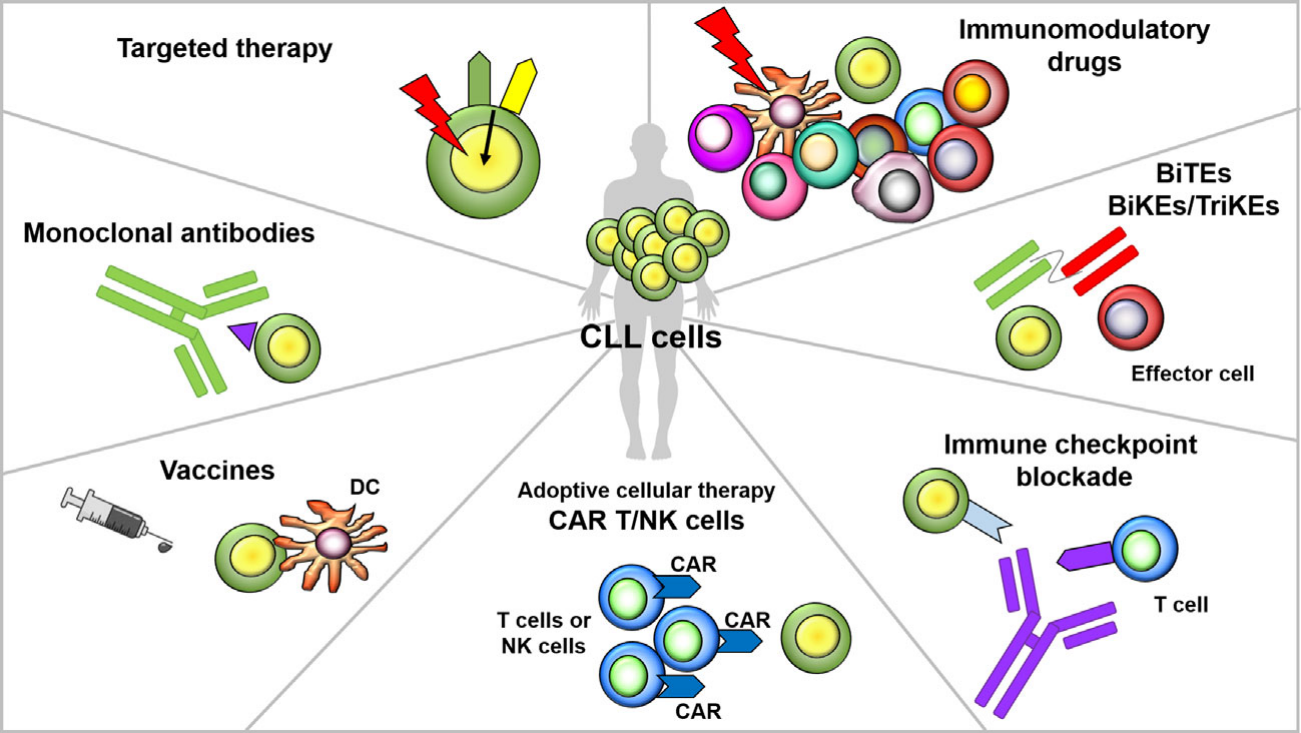
Overview of immunomodulatory agents and immune-based strategies in CLL. Several immunotherapeutic strategies are under evaluation in CLL. Targeted drugs, in addition to a direct anti-tumor activity, can exert off-tumor immunomodulatory effects on T cells and other immune elements. Immunomodulatory drugs (IMiDs) exert their activity through a broad immunomodulation and pleiotropic effects on multiple elements of the immune system (e.g, T, B, NK cells and DC). Monoclonal antibodies act through the recognition of a specific antigen expressed on the surface of tumor cells, which leads to the triggering of cytotoxic responses. Bi- and tri-specific killer cell engagers contain two or three antigen-recognition domains, and are designed to concomitantly target a tumor cell antigen and a molecule expressed on the surface of effectors cells, with the aim of directing immune cell activity toward malignant cells. Cancer vaccines aim at inducing tumor-specific T-cell immunity in an antigen-dependent fashion, thus eliciting immunological memory and long-term protection against cancer relapse. CAR T cells are autologous T lymphocytes engineered to express a chimeric receptor, which recognizes a tumor surface antigen; upon antigen recognition and co-stimulatory domains activation, a cytotoxic response is triggered, leading to tumor cells killing. Immune checkpoint inhibitors target the interactions between co-inhibitory receptors and their ligands thus avoiding the transmission of inhibitory signals that render T cells functionally exhausted.

The development of new targeted drugs has dramatically changed the treatment landscape of CLL. Despite their remarkable anti-tumor activity, agents like the B-cell receptor (BCR) signaling inhibitors and the Bcl-2 protein inhibitor venetoclax have some limitations, which include the development of drug resistance mechanisms and the less striking efficacy observed in patients carrying biological high-risk features [as reviewed in (156)]. The observation that allogeneic HSCT, which exploits immune-mediated graft-*versus*-leukemia effects to eradicate tumor cells, is the only treatment option with a long-term curative potential in CLL indicates that the immune system harbors the potential for curing the disease. Therefore, treatment strategies aimed at activating or exploiting effector arms of the autologous immune system to target tumor cells have been and currently are a focus of active investigations in CLL (as summarized in **Figure 2**). Here, we will give an overview of immune-based strategies that are currently used or explored in patients with CLL, thereby also providing an outlook on possible future therapeutic interventions.

### Agents With Broad Immunomodulatory Effects

In addition to their direct anti-tumor effect, new targeted drugs (i.e. BTK inhibitors, PI3K inhibitors, and the Bcl-2 inhibitor venetoclax) have demonstrated the ability to modulate non-neoplastic immune cell populations. PI3Kδ inhibition by idelalisib affects effector T-cell differentiation and functionality, and induces a disruption of Tregs suppressor activity, thus breaking immune tolerance both in CLL mice model and in patients (121, 124, 157). These evidences could explain the typical immune-mediated adverse events of the drug, mainly consisting in hepatotoxicity, enterocolitis, skin rash, and pneumonitis (157–159). Notably, idelalisib has also shown to positively impact the generation of T cells modified to express a CAR, when added to the culture during the manufacturing process, by inducing an enrichment of less-differentiated naïve-like T cells, a decrease in the expression of exhaustion markers, and a normalization of the CD4:CD8 ratio (160). These data suggest the possibility of exploiting the immunomodulatory effects of idelalisib during the preparation of adoptive immunotherapies, thus avoiding the adverse effects determined by drug administration to patients.

Immune changes exerted by ibrutinib have been partially characterized in CLL. Most studies report a normalization of T-cell population counts during treatment with ibrutinib, with the exception of the paper by Long *et al*., which shows an increase in CD4+ and CD8+ T-cell numbers. Moreover, an increase in TCR diversity repertoire and a decrease in the expression of T-cell exhaustion markers and immune checkpoints have been observed in CLL patients receiving ibrutinib (161–169). From the functional standpoint, preclinical data did not demonstrate a beneficial effect of ibrutinib on T cells (170). However ibrutinib-treated patients showed increased response rates to CAR T cells, thus supporting the potential benefit of combination strategies (171, 172). The immunomodulatory activity of ibrutinib is at least partially attributable to its off-target effects on tyrosine kinases other than BTK, such as the IL-2 inducible kinase (ITK), which is mainly expressed by T cells (173). However, at this stage, it is not completely elucidated whether ibrutinib immunomodulation is also connected to the reduction of tumor burden and to the suppression of tumor-microenvironment interactions, occurring during patients treatment. In line with this latter hypothesis is the observation that also second-generation BTK inhibitors (i.e. acalabrutinib and zanubrutinib), despite their higher selectivity and reduced off-target effects, still show some immunomodulatory properties (165, 174). Consistently, immune changes occurring during venetoclax treatment may be at least partially connected to a clinical response to therapy, driving the attenuation of leukemia-induced immune alterations. In particular, venetoclax has demonstrated to i) normalize B, T, and NK-cell count, ii) decrese the percentage of tumor-supportive T-cell subsets, iii) reduce the frequency of PD-1+ CD8+ T cells, and iv) impair NK-cell activation in CLL patients (114). Recent data also showed that venetoclax increases the anti-tumor effects of T-cell-based therapy against B-cell lines *in vitro* and in solid tumor-bearing mice model (175, 176).

Immunomodulatory drugs (IMiDs) have also been—and currently are—under evaluation for their anti-tumor effects in CLL. In the past 15 years, several clinical trials enrolling CLL patients have demonstrated the clinical efficacy of lenalidomide (**Table 1**), but have also highlighted its unique toxicity profile and a rate of adverse events that suggests caution in incorporating this agent in treatment strategies [as reviewed in (195)]. One aspect of lenalidomide that has been considered worth of investigation consists in its pleiotropic effects on the immune system. In fact, in spite of the absence of a direct *in vitro* cytotoxicity toward CLL cells, lenalidomide is able to favor the immune recognition of leukemic cells—through the upregulation of surface ligands and receptors—and to induce an indirect anti-tumor activity through the immunostimulation of DC as well as T, B, and NK cells. CLL patients treated with lenalidomide have a restored T-cell function, especially in terms of Th1-type cytokine release (IL-2, IFN-γ, TNF-α, IL-21), and formation of functional immune synapses (99, 155). In addition, lenalidomide induces wide-range immunomodulatory effects on different immune cell compartments, among which i) a decrease in Tregs count, ii) an increase in Th17 cell number, iii) an upregulation of the NKG2D activating receptor on NK cells, and iv) a rise in Ig production by normal polyclonal B cells. Besides the immune system re-education, treatment with lenalidomide also modulates interactions of CLL cells with NLC and stromal cells within the tumor microenvironment (155) [and reviewed in (195, 196)].

**Table 1:** Clinical trials evaluating the efficacy and toxicity of lenalidomide, used as a single agent or in combination regimens, for the treatment of patients with CLL.

Drug regimen	Setting	Efficacy	Toxicities	References
Lenalidomide single agent days 1–21 of 28-day cycles	Phase II 45 patients R/R CLL	ORR 47% 9% CR 1-year PFS 81%	G≥3 neutropenia 70% G≥3 thrombocytopenia 45% G≥3 anemia 18% G≥3 infections 5% G≥3 febrile neutropenia 15% Fatigue 73% Tumor flare reaction 58% TLS 5%	Chanan-Khan *et al.* (177)
Lenalidomide single agent continuously	Phase II 44 patients R/R CLL	ORR 32% 7% CR 14-month OS 73%	G≥3 neutropenia 41% G≥3 thrombocytopenia 15% G≥3 anemia 3% G≥3 infections 6%	Ferrajoli *et al.* (178) NCT00267059
Lenalidomide single agent days 1–21 of 28-day cycles	Phase II 25 patients Previously untreated CLL	ORR 56% 0 CR 2-year OS 92% PFS 89%	G≥3 neutropenia 72% G≥3 thrombocytopenia 28% Tumor flare reaction 88% Fatigue 72% Infections 60% Rash 64%	Chen *et al.* (179)
Lenalidomide single agent continuously	Phase II 60 elderly patients Previously untreated CLL/SLL	ORR 65% 10% CR 2-year PFS 60% 2-year OS 88%	G≥3 neutropenia 73% G≥3 thrombocytopenia 34% G≥3 anemia 15% G≥3 infections 10% Tumor flare reaction 27%	Badoux *et al.* (180) NCT00535873
Lenalidomide single agent continuously (different starting doses)	Phase II 103 patients R/R CLL	ORR 40% 8% CR; median PFS 9.7 months Median OS 33 months	G≥3 neutropenia 77% G≥3 thrombocytopenia 48% G≥3 anemia 4% G≥3 infections 41% Embolism 6% TLS 3%	Wendtner *et al.* (181) NCT00419250
Lenalidomide single agent continuously	Phase III 225 elderly patients Previously untreated CLL	ORR 55% 3% CR Median PFS 30.8 months	G≥3 neutropenia 49% G≥3 thrombocytopenia 25% G≥3 anemia 7% G≥3 infections 9% Tumor flare reaction 52% Rash 50%	Chanan-Khan *et al.* (182) NCT00910910
Lenalidomide and rituximab	Phase II 59 patients R/R CLL	ORR 66% 12% CR Median TTF 17.4 months 3-year OS 71%	G≥3 neutropenia 73% G≥3 thrombocytopenia 34% G≥3 anemia 15% G≥3 infections 15% Neutropenic fever 10%	Badoux *et al.* (183) NCT00759603
Lenalidomide and rituximab	Phase II 69 patients Previously untreated CLL	ORR 87% 14% CR Median PFS: 19–20 months	G≥3 neutropenia 58% Tumor flare 75% Fatigue 74% Transaminitis 65% Rash 61%	James *et al.* (184) NCT00628238
Lenalidomide and rituximab	Phase II 61 previously untreated + 59 R/R CLL	Previously untreated: ORR 73%; 35% CR; median PFS 50 months R/R: ORR 64%; 28% CR; median PFS 28 months	G≥3 neutropenia 53% G≥3 thrombocytopenia 23% G≥3 anemia 13% G≥3 infections 27% G≥3 cardiovascular 10% Second primary tumors 5%	Strati *et al.* (185) NCT01446133
Lenalidomide and ofatumumab	Phase II 21 patients R/R CLL	ORR 48% 0 CR Median OS 21.5 months	G≥3 neutropenia 47% G≥3 thrombocytopenia 71%Fatigue 52% Tumor flare reaction 43%	Costa *et al.* (186) NCT01123356
Lenalidomide and ofatumumab	Phase II 34 patients R/R CLL	ORR 71% 18% CR Median PFS 16 months 5-year OS 53%	G≥3 neutropenia 82% G≥3 thrombocytopenia 18% G≥3 anemia 6% G≥3 infections 60% Venous thromboembolysm 9%	Vitale *et al.* (187) NCT01002755
Lenalidomide, rituximab and fludarabine	Phase I 9 patients Previously untreated CLL	ORR 56% 11% CR	78% of patients stopped therapy because of toxicity (44% hematological toxicity)	Brown *et al.* (188)
Lenalidomide, rituximab and bendamustine	Phase I-II 22 patients Previously untreated and R/R CLL	ORR 50% 0 CR Median PFS 8 months	95% of patients had at least one G≥3 adverse event G≥3 neutropenia 71% G≥3 thrombocytopenia 19% G≥3 anemia 5% G≥3 infections 47%	Maurer *et al.* (189) NCT01558167
Lenalidomide, fludarabine and cyclophosphamide	Phase I-II 42 patients R/R CLL	ORR 62% 22% CR Median PFS 19 months Median OS 45 months	G≥3 neutropenia 65% G≥3 thrombocytopenia 17% G≥3 infections 7%	Mauro *et al.* (190) NCT00727415
Lenalidomide, rituximab and ibrutinib	Phase I 12 patients R/R CLL	ORR 67% 8% CR 1-year PFS 78%	G≥3 neutropenia 67% Diarrhea 58% Myalgia/arthralgia 58% Rash 42% G≥3 infections 25%	Ujjani *et al.* (191) NCT02200848
Lenalidomide, rituximab and chlorambucil, followed by lenalidomide consolidation	Phase I-II 63 elderly or unfit patients Previously untreated CLL	ORR after induction 83% (0 CR) ORR after consolidation 93% (14% CR) 2-year PFS 58%	During induction: G≥3 neutropenia 73% G≥3 thrombocytopenia 15% Rash 11% G≥3 infections 9%	Kater *et al.* (192) EudraCT 2010-022294-34
Lenalidomide, rituximab and bendamustine	Phase I 13 patients Previously untreated CLL	ORR 87% 39% CR	G≥3 neutropenia 52% G≥3 anemia 26% G≥3 thrombocytopenia 22% G≥3 febrile neutropenia 13% G≥3 rash 26%	Soumerai *et al.* (193) NCT01400685
Lenalidomide and dexamethasone	Phase II 31 patients Previously untreated CLL	ORR 74% 10% CR Median PFS 27 months	G≥3 neutropenia 61% Febrile neutropenia 19% Rash 65% Diarrhea 61% Edema 55%	Chen *et al.* (194) NCT01133743

CLL, chronic lymphocytic leukemia; CR, complete response; G≥3, grade ≥3; ORR, overall response rate; OS, overall survival; PFS, progression free survival; R/R, relapsed or refractory; SLL, small lymphocytic lymphoma; TLS, tumor lysis syndrome; TTF, time to treatment failure.

Compounds showing the same potent immunomodulatory effects as lenalidomide but characterized by a more manageable safety profile could represent an interesting therapeutic option for patients with CLL. Avadomide (CC-122) is a novel orally available pleiotropic pathway modulator, which has demonstrated enhanced anti-proliferative activities compared to lenalidomide, as well as the ability of eliciting anti-tumor T cell-mediated immune responses when combined with checkpoint inhibitors in preclinical models of CLL (197, 198). Based on these observations, a phase I/II clinical trial is currently evaluating the combination of avadomide plus ibrutinib or avadomide plus obinutuzumab in patients with CLL (NCT02406742).

### Monoclonal Antibodies

The targeting of tumor surface antigens with mAb has been the first form of immunotherapy broadly applied in the treatment of CLL. CD20, a transmembrane protein typically expressed on the B-cell surface, represents an ideal target for antibody-based therapy, and its targeting currently provides the best clinical results obtained with mAb therapy in CLL. Rituximab, which was approved by Food and Drug Administration (FDA) in 1998, is a chimeric mouse-human anti-CD20 mAb. The anti-tumor activity of rituximab is exerted through CDC and ADCC mechanisms, but is limited by the low-level of expression of the CD20 antigen on the surface of CLL cells, by the presence of circulating shed CD20 antigen interfering with the binding of the mAb to leukemic cells and by the selection of antigen loss variants in rituximab-treated patients (199–201). Based on its poor efficacy when used as a single agent, rituximab is mainly used in combination with other drugs for the treatment of CLL patients (202). Rituximab has demonstrated to improve the efficacy of chemotherapy agents, and the combinations FCR and bendamustine plus rituximab (BR) have been validated in phase III randomized clinical trials as the standard therapy for previously untreated, fit patients without *TP53* abnormalities (203–205). However, their use in the frontline setting has been recently challenged by studies comparing chemoimmunotherapy with targeted drugs, used alone or in combinations with rituximab, especially for the treatment of high-risk patients carrying unmutated IGHV and/or del(11q) (206, 207).

Next-generation anti-CD20 mAb with enhanced cytotoxic functions have later been developed. Ofatumumab is an example of type I (or rituximab-like) mAb, that mainly induces tumor cell lysis through CDC and ADCC [as reviewed in (208, 209)]. Obinutuzumab is a type II anti-CD20 mAb (i.e. inducing cytotoxicity mainly through programmed cell death and ADCC) characterized by an engineered structure that allows an increased recruitment of effector cells, a potentiated ADCC and a more efficient NK cell degranulation (210). Ofatumumab and obinutuzumab have shown efficacy in phase II and/or phase III clinical trials when used in combination with conventional chemotherapy (211, 212), BCR inhibitors (213–215), or venetoclax (216). More recently, ublituximab, another type I anti-CD20 mAb, has demonstrated clinical efficacy in CLL patients when administered alone or in combination with chemotherapy or targeted agents (217–219).

Other targets have been or currently are under evaluation for mAb-based therapeutic strategies in CLL. Among them, CD19 is particularly interesting, being a pan-B lymphocyte surface receptor, which is not expressed by hematopoietic stem cells and other immune cells, except for follicular DC [as reviewed in (220)]. CD19 is ubiquitously expressed on CLL cells and currently, results for two different anti-CD19 mAb evaluated in phase I studies are available. Tafasitamab is an anti-CD19 mAb with an engineered Fc region to enhance CD16 binding affinity, whereas inebilizumab is an affinity-optimized anti-CD19 mAb that enacts malignant clone elimination *via* ADCC. Both drugs have demonstrated safety and preliminary efficacy in previously treated CLL patients (221, 222). Tafasitamab containing regimens are currently under evaluation in two different phase II clinical trials enrolling patients with CLL (tafasitamab in combination with lenalidomide in the NCT02005289 trial and in combination with idelalisib or venetoclax in the NCT02639910 trial).

An additional target currently under investigation for CLL immunotherapy is CD37. CD37 is broadly and selectively expressed on tumor cells from B-cell malignancies, including CLL cells, where it is involved in various biological processes such as cell adhesion, proliferation, survival, and trafficking [as reviewed in (223)]. Otlertuzumab is an anti-CD37 fusion protein obtained from a chimeric protein (SMIP-016) and engineered to exhibit the full binding activity of an anti-CD37 mAb at one-third of the regular antibody size. The mechanism of action of otlertuzumab consists in the triggering of a direct pro-apoptotic effect and in the induction of ADCC, while sparing the activation of the complement system (224). Single-agent otlertuzumab has demonstrated a modest activity and an acceptable safety profile in a phase I study enrolling treatment-naïve and pre-treated CLL patients (225). In a following phase II trial, the same molecule in combination with bendamustine significantly increased the response rate and prolonged the progression free survival over single agent bendamustine in patients with relapsed or refractory CLL (226). An additional anti-CD37 mAb is BI 836826, which has been Fc-engineered to improve ADCC activity. In a phase I study it has shown an acceptable tolerability and a notable efficacy, being especially active in CLL patients with del(17p) and/or *TP53* mutations (227). BI 836826 is currently under evaluation for its safety and tolerability when given in combination with ibrutinib to patients with relapsed/refractory CLL (NCT02759016).

The ideal targets for a successful anti-tumor immunotherapy are tumor associated antigens (TAA), which are molecules with a unique or highly preferential expression on malignant cells and with a crucial role for the growth and survival of the tumor. In this context, the receptor tyrosine kinase-like orphan receptor 1 (ROR1) can be considered a putative TAA for CLL, being a cancer stem cell antigen almost exclusively expressed on tumor cells and involved in the biology and aggressiveness of the disease (228). Several immune-based strategies targeting ROR1 are currently being investigated, including mAb. Cirmtuzumab, an anti-ROR1 mAb, has demonstrated to be well tolerated and effective at inhibiting ROR1 signaling in a phase I study enrolling patients with progressive, refractory, and relapsed CLL (229), and is currently under evaluation for its safety and efficacy when given in combination with ibrutinib in patients with B-cell lymphoid malignancies in a phase Ib/II protocol (NCT03088878).

### Bi-Specific Antibodies and Bi- and Tri-Specific Killer Cell Engagers

Bi-specific antibodies (bsAb) combine specificities of two antibodies simultaneously, addressing different antigens or epitopes on the cell surface, and include a large family of molecules with different formats. Among bsAb, bi-specific T cells engagers (BiTEs) and bi- or tri- specific killer engagers (BiKE or TriKE) are dual or triple targeting antibodies, which act by simultaneously binding tumor antigens and effector cells (T cells or NK cells), thus leading to the creation of a new immunological synapse and the triggering of cytotoxic responses. Of note, TriKEs recognize two different antigens on tumor targets allowing the binding of cancer cells even when one antigen is lost, and thus avoiding the occurrence of escape variants [as reviewed in (230–232)].

Blinatumomab, a CD19/CD3 bsAb designed in the BiTE format, was the first bsAb studied in setting of CLL patients. In B-cell acute lymphoblastic leukemia (B-ALL), where it is currently approved for the treatment of patients with a relapsed/refractory disease or not achieving an undetectable minimal residual disease, blinatumomab has shown a good anti-leukemic activity associated with a low treatment-related mortality (233). Blinatumomab has demonstrated to effectively kill CLL cells *in vitro*, through the induction of proliferation, cytokine production and granzyme B secretion in autologous T cells. As demonstrated by Wong *et al.*, the formation of immunological synapses between T cells and CLL cells induced *in vitro* by blinatumomab indicates that this CD19/CD3 BiTE is able to overcome the T-cell dysfunction frequently observed in CLL patients (234). In preclinical studies, blinatumomab induced cytotoxicity against tumor cells at very low T-cell:tumor cell ratios, in samples from both treatment-naïve and treated patients, and also in the presence of pro-survival signals (234). A phase I study has demonstrated the feasibility of blinatumomab in relapsed/refractory B-cell non-Hodgkin lymphomas, including small lymphocytic lymphoma (235), and a clinical trial is currently testing the association between lenalidomide and blinatumomab in the same clinical setting (NCT02568553). However, specific data on the tolerability and efficacy of blinatumomab in the CLL patient population are not available.

The main limitation to the efficacy of BiTE constructs is their short half-life that requires these drugs to be administered continuously. To overcome structural limitations of BiTEs, and specifically their poor stability, next-generation bsAb with a more favorable pharmacokinetic profile are currently under investigation. To this aim, a new bsAb platform represented by dual affinity re-targeting (DART) molecules was developed. The MGD011 CD3xCD19 DART (also known as JNJ-64052781) has demonstrated a good *in vitro* efficacy in killing CLL cells by recruiting CLL-derived T cells against the tumor. MGD011 has shown the ability to induce activation and proliferation of T cells from CLL patients, and to promote a partial restore of their immunological disfunctions. Interestingly, MGD011 is also able to kill venetoclax-resistant CLL cells, through a mechanisms that is independent from Bcl-2-mediated apoptosis (236, 237).

Another recently-developed CD19/CD3 bsAb, designed in the single-chain Fv-Fc format (CD19/CD3-scFv-Fc), has shown to induce a particularly rapid killing of CLL cells isolated from ibrutinib-treated patients, including those with acquired ibrutinib-resistance (238).

Although not fully understood, this increased cytotoxicity seems at least in part attributable to an improved performance of T cells isolated from patients treated with ibrutinib. Consistently, a BiTE targeting ROR1 has shown an enhanced cytotoxic activity against primary leukemic cells when used in the presence of T cells isolated from ibrutinib-treated CLL patients (239). Therefore, all these data highlight the importance of the reversal of CLL-related T-cell impairment to improve the BiTE activity.

Another strategy to bypass the T-cell impairment is to exploit engagers designed to target the activity of effector cells of the innate immune system. To date, in CLL, BiKEs and TriKEs engaging NK cells have been exclusively studied in the preclinical setting, but available data encourage their potential translation to the clinic. The therapeutic potential of a CD16/CD19 BiKE and a CD16/CD19/CD22 TriKE has already been demonstrated in a preclinical study showing their ability to trigger NK cell functions in terms of cytokine and chemokine production, secretion of lytic granules and induction of tumor cell death (240). TriKEs recognizing the NKG2D receptor ligand ULBP2 (ULBP2/aCD19/aCD19 and ULBP2/aCD19/aCD33 TriKEs) have also been used to activate NK cells and showed a superior *in vitro* and *in vivo* anti-tumor activity against CLL compared to the bi-specific counterparts (201).

### Tumor Vaccines

The ultimate goal of a cancer vaccine is the activation and expansion of cytotoxic T lymphocytes against tumor targets, thus promoting the elimination of the tumor and inducing a long-term protection against possible relapses. In CLL, the main obstacles for the production of effective tumor vaccines are the difficulty in the selection of an ideal TAA, which should be specific for the tumor but broadly expressed in the patients population, and the presence of immune dysfunctions limiting the triggering of effective and peristent anti-tumor responses.

To overcome the difficulties connected to the identification of an optimal TAA, vaccine formulations consisting of autologous whole tumor cells genetically modified to express cytokines or costimulatory molecules have been tested. The manipulation of autologous CLL cells to express a functional ligand for the CD40 molecule (i.e. CD40L or CD154) has shown to upregulate costimulatory factors on leukemic cells and to induce the generation of cytotoxic T lymphocytes capable of specifically recognizing parental non-modified leukemic cells *in vitro* (241). Based on these preclinical results, Wierda *et al.* have designed a phase I study to explore the clinical efficacy of a cancer vaccine consisting of autologous CLL cells genetically modified to express a human form of the CD40L molecule. Results from this study demonstrated that this vaccine formulation is well tolerated, has biological and clinical activity, and may enhance the susceptibility of CLL cells with del(17p) to subsequent chemoimmunotherapy (242). Interestingly, the preclinical use of genetically modified CD40L-expressing CLL cells in combination with IL-2- or OX40L-expressing CLL cells has shown to produce an even more pronounced T-cell activation and trigger therapeutically significant leukemia-specific immune responses (243, 244). Despite the encouraging preliminary results, all the tested tumor cell-based vaccine formulations failed to produce reproducible clinical effects, mainly due to the existence of immune escape mechanisms and deep CLL-driven defects in the immune system. One approach aiming at restoring the immune competence of CLL patients is the use of checkpoint-blockade inhibitors to “release” the immune system to target cancer cells. In this context, the use of a tumor vaccine consisting of irradiated autologous tumor cells coated with an antibody targeting the CD200 immunoregulatory molecule has shown to be effective in a xenogenic model of CLL (245).

An alternative strategy to induce effective anti-tumor responses is the use of vaccine formulations exploiting the antigen presentation ability of DC. The feasibility and safety of a vaccine consisting of DC loaded with apoptotic bodies derived from autologous CLL cells has already been demonstrated in an early-phase clinical trial (74). The lack of meaningful clinical effects, in this as in most other CLL vaccination trials, illustrates the need to identify more potent immune adjuvants for CLL. To this aim, lenalidomide, administered in combination with a DC vaccine, has shown its ability to elicit tumor-specific T-cells responses, although in the presence of relevant autoimmune compications that suggest caution in further exploring this drug as an immune adjuvant in CLL (75).

### Cellular Immunotherapy and CAR T Cells

Adoptive cellular immunotherapy is an alternative approach to exploit the immune system to fight tumors, and consists in the isolation and expansion of effector cells that are then transferred to patients.

NK cells and γδ T cells are particularly appealing candidates for cellular immunotherapy, thanks to their peculiar ability of recognizing and targeting tumor cells in an MHC unrestricted manner, which favors the induction of effective allogeneic and autologous anti-tumor responses [as reviewed in (246–248)]. In the setting of CLL, Almeida *et al.* designed a protocol for the clinical grade expansion and the preclinical testing of cytotoxic Vδ1+ T cells, named Delta One T (DOT) cells. DOT cells express NCRs, which synergize with the TCR to mediate leukemic cell targeting *in vitro*, and inhibit tumor growth in xenograft models of CLL (67).

Immune effector cells may also be genetically engineered with the aim of improving and specifically directing their killing properties against the tumor. CAR T cells have been under development for more than 30 years (249), and recently entered the therapeutic armamentarium for lymphoproliferative diseases [as reviewed in (250–252)]. Anti-CD19 CAR T cells are currently approved by FDA and European Medicine Agency for the treatment of patients with aggressive B-cell lymphomas or B-ALL. In other hematological malignancies, including CLL, several challenges still need to be overcome for successful application of CAR T-cell therapies, including identifying alternative or additional target antigens and reversing repressive tumor microenvironments that hamper CAR T-cell function.

Novel CAR constructs that target antigens other than CD19 (e.g. CD20, CD22, ROR1) or that concomitantly target more than one antigen (e.g. CD19 and CD20, CD19 and CD22) are currently under evaluation in early phase clinical studies. Clinical trials evaluating CAR-based cellular therapies in patients with CLL are listed in **Tables 2** and **3**.

**Table 2:** Clinical trials evaluating the efficacy and toxicity of CAR T- and CAR NK-cell treatment in CLL patients.

Drug regimen	Setting	Efficacy	Toxicities	References
Autologous anti-CD19 CAR T cells	3 patients R/R CLL	ORR 100% 67% CR	CRS 100% CRES 0	Kalos *et al.* (253) NCT01029366
Autologous anti-CD19 CAR T cells	14 patients R/R CLL	ORR 57%28% CR	CRS 64% CRES 36%	Porter *et al.* (254) NCT01029366
Autologous anti-CD19 CAR T cells + aldesleukin 720000 UI/kg every 8 hours	4 patients R/R CLL	ORR 75% 25% CR	CRS 100% CRES 0	Kochenderfer *et al.* (255) NCT00924326
Autologous anti-CD19 CAR T cells	5 patients (1 Richter syndrome) R/R CLL	ORR 100% 60% CR	CRS 60% CRES 20%	Kochenderfer *et al.* (256) NCT00924326
Allogeneic anti-CD19 CAR T cells	4 patients (2 Richter syndrome) R/R CLL after allogeneic HSCT	ORR 25% 25% PR	CRS ND CRES ND	Cruz *et al.* (257) NCT00840853
Autologous anti-CD19 CAR T cells + ibrutinib for ≥1 year	3 patients R/R CLL	ORR 100% 33% CR	CRS ND CRES ND	Fraietta *et al.* (258) NCT01747486 NCT01105247 NCT01217749
Autologous anti-κ light chain CAR T cells	2 patients R/R CLL	ORR 0	CRS ND CRES ND	Ramos *et al.* (259) NCT00881920
Allogeneic anti-CD19 CAR T cells	5 patients R/R CLL	ORR 40% 20% CR	CRS 80% CRES 0	Brudno *et al.* (260)
Autologous anti-CD19 CAR T cells	13 patients R/R CLL post ibrutinib	ORR 83% 50% CR	CRS ND CRES ND	Turtle *et al.* (261) NCT01865617
Autologous anti-CD19 CAR T cells	24 patients (5 Richter syndrome) R/R CLL	ORR 67% 17% CR	CRS 83% CRES 33%	Turtle *et al.* (262) NCT01865617
Autologous anti-CD19 CAR T cells	8 patients PR CLL after first-line treatment with pentostatin, cyclophosphamide and rituximab	ORR 25% 25% CR	CRS 50% CRES 0	Geyer *et al.* (263) NCT01416974
Autologous anti-CD19 CAR T cells	19 patients R/R CLL to 6 month-therapy with ibrutinib	ORR 71% 43% CR	CRS 95% CRES 26%	Gill *et al.* (172) NCT02640209
Autologous anti-CD19 CAR T cells	2 patients R/R CLL	ORR 50% 50% CR	CRS 0 CRES 0	Enblad *et al.* (264) NCT02132624
Autologous anti-CD19 CAR T cells	1 patient R/R CLL	ORR 0 100% SD	CRS ND CRES ND	Ramos *et al.* (265) NCT01853631
Autologous anti-CD19 CAR T cells	10 patients R/R CLL	ORR 75% 50% CR	CRS 80% CRES 30%	Siddiqi *et al.* (266) NCT03331198
Autologous anti-CD19 CAR T cells	22 patients R/R CLL after 2 or 3 lines of therapy including ibrutinib	ORR 82% 45% CR	CRS 9% CRES 23%	Siddiqi *et al.* (267) NCT03331198
Autologous anti-CD19 CAR T cells + ibrutinib 420 mg daily for 2 weeks (days −50 to −36)	1 patient R/R CLL	ORR 100% 100% CR	CRS 100% CRES 0	Delgado *et al.* (268) NCT03144583
Autologous anti-CD19 CAR T cells	8 patients R/R CLL	ORR 12% 12% PR 37% SD	CRS 100% CRES 0	Brentjens *et al.* (269) NCT00466531
Autologous anti-CD19 CAR T cells (5 patients received ibrutinib at the time of T-cell collection and/or CAR T-cell administration)	16 patients R/R CLL	ORR 37% 12% CR MRD – 6% CR MRD +	CRS 100% CRES 37%	Geyer *et al.* (270) NCT00466531
Autologous anti-CD19 CAR T cells	2 patients R/R CLL	ND	CRS ND CRES 0	Schubert *et al.* (271) NCT036765041
Anti-CD19 CAR T cells	13 patients (3 Richter syndrome) R/R CLL	42% CR	CRS 85% CRES 85%	Batlevi *et al.* (272)^1^ NCT030851731
Anti-CD19/CD20 CAR T cells	2 patients R/R CLL	ORR 82% 54% CR	CRS 54% CRES 27%	Shah *et al.* (273)^1^ NCT03019055
Autologous anti-CD19 CAR T cells + ibrutinib from at least 2 weeks prior to leukapheresis until at least 3 months after CAR T infusion *vs* autologous anti-CD19 CAR T cells alone	19 patients *vs* 30 (4 Richter syndrome)	ORR 83% 22% CR 72% MRD – assessed by cytometry 61% MRD - assessed by *IGH* sequencing *vs* ORR 56% 67% MRD – assesed by cytometry 60% MRD - assesed by *IGH* sequencing	CRS 74% *vs* 95% CRES 26% *vs* 42%	Gauthier *et al.* (150) NCT01865617
Umbilical cord blood derived anti-CD19 CAR NK	5 patients (1 Richter) R/R CLL	3/5 ORR 3 CR	CRS 0 CRES 0	Liu *et al.* (274) NCT03056339

CLL, chronic lymphocytic leukemia; CR, complete remission; CRES, CAR T cell-related encephalopathy syndrome; CRS, cytokine release syndrome; HSCT, hematopoietic stem cell transplantation; MRD, minimal residual disease; ND, no data; ORR, overall response rate; PR, partial response; R/R relapsed or refractory disease; SD, stable disease.

^1^Data for CLL patients only non-available.

**Table 3:** Ongoing clinical trials evaluating CAR T-cell treatment in CLL patients.

NCT number	Drug regimen	Setting
NCT03881774	Cord blood derived CAR T cells	R/R CLL after autologous CAR T cells therapy or who fail to preparation for autologous CAR T cells
NCT04271410	Anti-CD19 CAR T cells	R/R CLL
NCT04156243	Anti-CD19 CAR T cells	R/R CLL
NCT04014894	Anti-CD19 CAR T cells	R/R CLL
NCT04271800	Anti-CD19 CAR T cells	R/R CLL
NCT03685786	Anti-CD19 CAR T cells + autologous HSCT	MRD+ CLL
NCT03960840	Autologous anti-CD19 CAR T cells	SD or PR CLL after 6-month therapy with ibrutinib or R/R CLL
NCT02963038	Autologous anti-CD19 CAR T cells	R/R CLL
NCT03110640	Autologous anti-CD19 CAR T cells	R/R CLL
NCT03302403	Autologous anti-CD19 CAR T cells	R/R CLL
NCT03579888	Autologous anti-CD19 CAR T cells	R/R CLL who have undergone allogeneic HSCT
NCT02153580	Autologous anti-CD19 CAR T cells	R/R CLL
NCT03050190	Autologous anti-CD19 CAR T cells	R/R CLL
NCT03624036	Autologous anti-CD19 CAR T cells	R/R CLL
NCT03853616	Autologous anti-CD19 CAR T cells	R/R CLL
NCT03383952	Autologous anti-CD19 CAR T cells	R/R CLL
NCT03191773	Autologous anti-CD19 CAR T cells	R/R CLL
NCT03166878	Allogeneic anti-CD19 CAR T cells	R/R CLL
NCT03277729	Autologous anti-CD20 CAR T cells	R/R CLL
NCT00621452	Autologous anti-CD20 CAR T cells	R/R CLL
NCT00012207	Autologous anti-CD20 CAR T cells	R/R CLL
NCT01735604	Autologous anti-CD20 CAR T cells	R/R CLL
NCT04030195	Allogeneic anti-CD20 CAR T cells	R/R CLL
NCT02794961	Autologous anti-CD22 CAR T cells	R/R CLL
NCT02194374	Autologous anti-ROR1 CAR T cells	R/R or untreated CLL with del17p and not eligible for allogeneic HSCT
NCT02706392	Autologous anti-ROR1 CAR T cells	R/R CLL
NCT04156178	Anti-CD19/CD20 CAR T cells	R/R CLL
NCT04260945	Anti-CD19/CD20 CAR T cells	R/R CLL
NCT04007029	Autologous anti-CD19/CD20 CAR T cells	R/R CLL
NCT03097770	Autologous or allogenic anti-CD19/CD20 CAR T cells	R/R CLL
NCT03398967	Allogeneic anti-CD19/CD20 or anti-CD19/CD22 CAR T cells	R/R CLL
NCT04029038	Autologous anti-CD19/CD22 CAR T cells	R/R CLL
NCT03185494	Autologous or allogenic anti-CD19/CD22 CAR T cells	R/R CLL
NCT03125577	Autologous anti-CD19 and anti-CD20/CD22/CD30/CD38/CD70/CD123 CAR T cells	R/R CLL

CLL, chronic lymphocytic leukemia; HSCT, hematopoietic stem cell transplantation, MRD, minimal residual disease; PR partial response; R/R relapsed or refractory; SD, stable disease.

In spite of a safety profile not dissimilar to that observed in the setting of other lymphoproliferative diseases, which includes cytokine release syndrome (CRS) and CAR T cell-related encephalopathy syndrome (CRES) as major complications, to date, the efficacy results of CAR T cells in CLL have been relatively discouraging, mainly due to T-cell alterations paralleling disease evolution and hampering effective anti-tumor functions of autologous CAR T cells. Fraietta *et al.* demonstrated that the composition of the cellular product and the intrinsic T-cell functional fitness may have an impact on the therapeutic efficacy of CAR T cells in CLL. They showed that the ability of CAR T cells to expand during the manufacturing process is a predictor of response and correlates with *in vivo* proliferation, which is in turn responsible for a sustained anti-tumor activity. In addition to that, they observed that CAR T cells derived from patients who respond to the treatment are enriched in memory-cell lymphocytes, with enhancement of IL-6/STAT3 signals and STAT3-related cytokine production, whereas CAR T cells from patients who do not respond upregulate genetic programs involved in effector differentiation, glycolysis, exhaustion, and apoptosis (275).

A possible strategy to improve the clinical benefit of CAR T-cell therapy is the co-administration of targeted anti-tumor agents selected for their ability to exert immunomodulatory properties, with the aim of overcoming tumor-induced immune dysfunctions. Based on preclinical studies showing that ibrutinib could improve the anti-tumor efficacy of CAR T cells, a phase I clinical trial was conducted showing the safety and feasibility of ibrutinib administered in combination with anti-CD19 CAR T cells in relapsed and refractory CLL patients (171). In line with these results, recent preclinical data show that also the novel BTK inhibitor acalabrutinib can improve the *in vitro* and *in vivo* anti-tumor functions of CD19-directed CAR T cells (276).

Besides T cells, NK cells could represent a valid cellular carrier for CAR constructs. CAR NK cells have the advantage to be activated not only by the CAR target antigen, but also by NCRs, thus adding ADCC-mediated mechanisms to the CAR-mediated cell lysis. Due to the dysregulation of patient-derived NK cells, most studies addressing the efficacy of NK cells-based adoptive immunotherapy consisted in the transfer of *ex vivo* expanded allogeneic NK cells derived from healthy donors’ peripheral blood, umbilical cord blood, or cell lines (277, 278). Allogeneic NK cells can be safely used as effector cells since they do not require a full HLA-matching and they do not induce graft-*versus*-host disease, while harboring strong graft-*versus*-leukemia effects (279, 280). Cord blood-derived anti-CD19 CAR NK cells showed good activity towards CLL cells *in vitro*, and preliminary evidences from a clinical trial have already demonstrated the safety and efficacy of this approach in patients with CD19-positive tumors, including CLL (274, 281). Thanks to these reasons, CAR NK cells can be produced in allogeneic settings and easily used as an “off-the-shelf” treatment (26).

### Immune Checkpoint Inhibitors

The targeting of immune checkpoint molecules with the aim of reactivating the T-cell immune responses against tumor cells is an appealing therapeutic strategy. In the context of solid tumors, the blockade of immune checkpoint receptors or their cognate ligands by mAb has brought significant benefits for patients [as reviewed in (282)]. In CLL, encouraging preclinical results have been obtained in the studies that evaluated PD-1/PD-L1 axis disruption (283, 284). Specifically, *in vivo* treatment with an anti-PD-L1 antibody prevented the development of CLL in the Eµ-TCL1 mice model, also normalizing T cells and myeloid cell populations, and restoring T-cell functions (283). In the same murine model, it has been demonstrated that antibodies targeting PD-L1, but not PD-1, enhance the anti-tumor activity of ibrutinib treatment (170, 284). Notably, Wierz *et al*. have shown, in the Eµ-TCL1 mice model, that the antibody-based dual targeting of PD-1 and LAG3—but not the single targeting of PD-1—effectively limits the tumor development and restores different immune cell populations (284). These data support the concept that the simultaneous inhibition of different immune checkpoint molecules may represent an interesting therapeutic approach, which is worth to be explored.

Unfortunately, so far, disappointing results emerged from clinical trials evaluating immune checkpoint inhibitors in CLL, indicating that these compounds used as single agents are not sufficient to control the disease (**Table 4**). By contrast, interesting preliminary results in terms of response rate have been obtained when checkpoint inhibitors were administered, alone or in association with ibrutinib, to CLL patients developing a Richter’s transformation to diffuse large B-cell lymphoma, and particularly to those patients showing a higher expression of PD-L1 and PD-1 in the tumor microenvironment (285, 289). These data encourage further studies exploring the efficacy of checkpoint inhibitors in this setting, which still represents a significant unmet clinical need.

**Table 4:** Clinical trials evaluating the efficacy and toxicity of immune checkpoint inhibitors, used as single agents or in combination regimens, for the treatment of patients with CLL.

Drug regimen	Setting	Efficacy	Toxicities	References
Pembrolizumab every 3 weeks for up to 2 years	Phase II: 25 patients (9 Richter syndrome) R/R CLL	ORR 16% 1% CR Median PFS 3 months Median OS 10.7 months	G≥3 neutropenia 20% G≥3 thrombocytopenia 20% G≥3 anemia 20% G≥3 dyspnea 8% G≥3 hypoxia 8% G≥3 fatigue 8% G≥3 febrile neutropenia 8% G≥3 fever 4% G≥3 maculopapular rash 4% G3 lung infection 12% G3 hepatic toxicity 8% G5 sepsis 4%	Ding *et al.* (285) NCT02332980
Nivolumab 3 mg/kg IV every 2 weeks and ibrutinib 420 mg/day	Phase II 8 patients (4 Richter sydrome) R/R CLL *vs* 3 patients PR CLL to 9-month ibrutinib therapy	ORR 75% 50% PR *vs* ORR 100% 100% PR	No G≥3 AEs	Jain *et al.* (286) NCT02420912
Pidilizumab	Phase I 3 patients R/R CLL	ORR 0 67% SD	No G≥3 AEs	Berger *et al.* (287)
Samalizumab once every 28 days until progression or toxicity	Phase I 23 patients R/R CLL	ORR 4% 4PR	G≥3 hematological AEs 12% G≥3 reduced visual acuity 4% G≥3 muscular weakness 4% G≥3 RSV infection 4% G≥3 rash 4%	Mahadevan *et al.* (288)^1^ NCT00648739
Ibrutinib (420 mg or 560 mg) and nivolumab	Phase I-IIa 56 patients (20 Richter syndrome)	ORR 97%	G≥3 neutropenia 28% G≥3 anemia 23% G≥3 rash 8% G≥3 increased ALT 2% Serious AEs: anemia 4% and pneumonia 4%	Younes *et al.* (289)^1^ NCT02329847
For CLL: induction with umbralisib and ublituximab, consolidation with pembrolizumab, umbralisib and ublituximab, maintenance with umbralisib until progression or unacceptable AE. For Richter syndrome: umbralisib, ublituximab and pembrolizumab	Phase I/II 14 patients (5 Richter syndrome) R/R CLL	CLL: ORR 89% Richter syndrome: ORR 50%, 50% CR PFS 71% (median follow-up 15 months)	G≥3 neutropenia 43% G≥3 ALT/AST increase 21% G≥3 hypophosphatemia 21% G≥3 pneumonitis 7%	Mato *et al.* (290)
Nivolumab *vs* pembrolizumab and ibrutinib in 3 patients and venetoclax in 1 patient	7 *vs 3* patients R/R CLL (Richter syndrome)	ORR 10%	ND	Rogers *et al.* (291)
Ipilimumab and lenalidomide	Phase II 2 patients R/R CLL to allogeneic HSCT	ORR 0	G≥3 neutropenia 40% G2 anemia 40% G2 thrombocytopenia 20% Flare of GVHD 10% G2 nausea 10% G2 headache 10% G2 diarrhea 10%% G2 elevated transaminase 10% G2 hypertension 10% G2 hypothyroidism 10%	Khouri *et al.* (292)^1^ NCT01919619
Nivolumab single agent until progression or unacceptable toxicity, with planned deescalation based on toxicity	Phase I 1 patient R/R CLL to allogeneic HSCT	ORR 32% 1-year PFS 23%, 1-year OS 56%	G≥3 thrombocytopenia 14% G≥3 neutropenia 14% G≥3 anemia 11% G≥3 febrile neutropenia 11% G≥3 fatigue 7.1% G≥3 rash 3.6% G≥3 transaminitis 7.1% G≥3 fever 3.6% G≥3 lipase elevation 14% G≥3 pneumonitis 3.6% G≥3 abdominal pain 3.6% G≥3 nausea 3.6% G≥3 arthralgia 3.6% G≥3 bilirubin elevation 11% G 5 acute GVHD (liver and gut) 7.1% G 5 APS complicated by thrombotic CVA 3.6% G 5 sepsis with ARDS 3.6%	Davids *et al.* (293)^1^ NCT01822509
Umbralisib and pembrolizumab	Phase I R/R CLL	ND	ND	NCT03283137
Pembrolizumab	Phase II R/R CLL with Richter Syrndrome	ND	ND	NCT02576990
Ublituximab and umbralisib in combination with targeted immunotherapy	Phase I R/R CLL or Richter syndrome	ND	ND	NCT02535286
Ibrutinib, fludarabine and pembrolizumab	Phase II high-risk or R/R CLL/SLL	ND	ND	NCT03204188
Atezolizumab, obinutuzumab and venetoclax	Phase II CLL, SLL, R/R Richter syndrome	ND	ND	NCT02846623
Durvalumab monotherapy and in combination (with lenalidomide and rituximab; with ibrutinib; with bendamustine and rituximab)	Phase I-II lymphoma or CLL	ND	ND	NCT02733042
Anti-LAG-3 (BMS-986016) single agent and in combination with nivolumab	Phase I/IIa R/R CLL	ND	ND	NCT02061761

AE, adverse events; APS, anti-phospholipid antibody syndrome; ARDS, acute respiratory distress syndrome; CLL, chronic lymphocytic leukemia; CR, complete response; CVA, cerebral vascular accident; G≥3, grade ≥3; GVHD, graft-versus-host disease; HSCT, hematopoietic stem cell transplantation; ND, no data; ORR, overall response rate; OS, overall survival; PFS, progression free survival; PR, partial response; R/R, relapsed or refractory; SD, stable disease; SLL, small lymphocytic lymphoma.

^1^Data for CLL patients only non-available.

## Conclusions

It is well recognized that, in addition to the direct targeting of malignant cells, the disruption of the immune-tolerant microenvironment and the repair of immune system’s defects are necessary steps for disease control. With the aim of harnessing immune responses against tumor cells, during the years, different types of immune-based strategies have been developed and evaluated in CLL. Despite promising preclinical observations, results from pilot clinical studies have been often suboptimal in terms of long-term tumor control, mainly because they were obtained in patients with advanced-stage disease and who had been already heavily pre-treated. In CLL, several observations demonstrate that the tumor negatively affects the host immune system, which progressively accumulates dysfunctions contributing to disease progression. Therefore, in disease like CLL, characterized by a long-acting evolution and the accumulation of immunologic dysfunction, one possibility to improve the efficacy of immunotherapy could be its earlier positioning in the treatment sequencing. By doing this, the development of immune defects could be prevented and subsequent therapies could act in concert with the patient’s immune system against the tumor. However, the application of immunotherapy earlier in the course of the disease has to take into account the potential toxicities and the meaningful costs, and should be considered a valuable option only for patients with a high-risk disease and poor prognosis, who benefit less from currently available therapies.

An alternative strategy to improve patients’ outcome is the identification of optimal combination treatments targeting both the CLL and the immune system, in order to reshape the functionality of the latter and properly address its reaction toward the tumor. Ibrutinib, and to some extent also next-generation BTK inhibitors and venetoclax, have shown to improve the host T-cell functions. Therefore, these targeted drugs, as well as other agents with more broad immunomodulatory properties—such as IMiDs and checkpoint inhibitors—are currently under evaluation for their ability to potentiate the efficacy of other immunotherapeutic strategies. In this context, combination trials of ibrutinib and T-cell directed immunotherapies, such as anti-CD19 CAR T cells, have already provided promising results and support the potential of this approach.

## Author Contributions

VG and FP reviewed the literature and wrote the manuscript. VG and FP equally contributed to this work. CS and EB contributed to literature review. MB contributed to manuscript revision. CV and MC designed the review and revised the manuscript. CV and MC equally contributed to this work. All authors contributed to the article and approved the submitted version.

## Funding

This research received no external funding. VG is a recipient of a fellowship from the “Associazione Damiano per l’Ematologia”.

## Conflict of Interest

MB has received honoraria from Sanofi, Celgene, Amgen, Janssen, Novartis, Bristol-Myers Squibb, and AbbVie; has served on the advisory boards for Janssen and GSK; has received research funding from Sanofi, Celgene, Amgen, Janssen, Novartis, Bristol-Myers Squibb, and Mundipharma. CV has received consultancy fees from Janssen. MC received honoraria from Janssen, Gilead, Abbvie, Shire and research support from Janssen and Karyopharm Therapeutics.

The remaining authors declare that the research was conducted in the absence of any commercial or financial relationships that could be construed as a potential conflict of interest.

The reviewer AK declared a past co-authorship with several of the authors CV and MC to the handling editor.
